# A Manual of General Pathology

**Published:** 1899-06

**Authors:** 


**A Manual of General Pathology.** By WALTER SYDNEY LAZARUS-BARLOW, M.D. Pp. xi., 795. London: J. & A. Churchill. 1898.

Pathological anatomy is not touched in this book, which deals solely with experimental and general pathology. There is no doubt that the English text-books on pathology have in the past confined themselves too exclusively to pathological anatomy, and the sections devoted to general pathology have been comparatively brief and unsatisfactory. They have, therefore, failed to keep pace with the advances made in experimental pathology during recent years, and thus to give a satisfactory account of a subject which has made great strides. To deal with the whole of pathology, general and anatomical, and at the same time to give an adequate account of those researches and conflicting views, which cannot be omitted without endangering the usefulness of the work, is however no longer possible in a text-book of anything like moderate dimensions, and there is therefore much to be said in favour of the author's contention that it is more convenient to consider general pathology in a separate treatise. Thus this volume comes naturally from the aspect from which it views pathology to occupy a place not hitherto satisfactorily filled by other text-books, and in this way performs a distinctly useful function, and seems likely to have a successful future before it. The manner in which the author has fulfilled his task deserves success, for the book is well written, and gives a very good account of all the most important advances in our knowledge of general pathology, and is brought thoroughly up to the level of the most recent researches. From the author's own investigations into the pathology of the blood-plasma, œdema, and absorption, it would be expected that the chapter on this difficult and at present much-debated subject would be a particularly good one, and there is an extremely able account of it, fully representative of present knowledge, and forming a distinctive feature of the work. The book is one to be recommended as a trustworthy one for students, and will also be found very valuable to practitioners who desire to keep their knowledge of general pathology not only up to date, but to extend it.

